# Elevated serum alkaline phosphatase and cardiovascular or all-cause mortality risk in dialysis patients: A meta-analysis

**DOI:** 10.1038/s41598-017-13387-z

**Published:** 2017-10-16

**Authors:** Yu Fan, Xin Jin, Menglin Jiang, Na Fang

**Affiliations:** Institute of Molecular Biology & Translational Medicine, the Affiliated People’s Hospital, Jiangsu University, (212002) Zhenjiang, Jiangsu PR China

## Abstract

Studies on serum alkaline phosphatase (ALP) and mortality risk in patients with end-stage renal disease (ESRD) undergoing dialysis have yielded conflicting results. This meta-analysis was designed to assess the association of serum ALP levels with cardiovascular or all-cause mortality risk among patients on dialysis. PubMed and Embase databases were searched until March 2017 for studies evaluating the association of serum ALP levels and cardiovascular or all-cause mortality risk in adult patients with ESRD undergoing maintenance hemodialysis or chronic peritoneal dialysis. Twelve studies enrolling 393,200 patients on dialysis were included. Compared with the reference low serum ALP category, pooled adjusted hazard risk (HR) of all-cause mortality was 1.46 (95% confidence interval [CI] 1.30–1.65) for patients on hemodialysis and 1.93 (95% CI 1.71–2.17) for peritoneal patients on dialysis. In addition, elevated serum ALP significantly increased cardiovascular mortality among patients on peritoneal dialysis (HR 2.39; 95% CI 1.23–4.65) but not in patients on hemodialysis (HR 1.08; 95% CI 0.84–1.40). Elevated serum ALP was an independent risk factor for all-cause mortality among patients on hemodialysis or peritoneal dialysis. Further well-designed prospective studies are needed to investigate the association of high serum ALP levels with cardiovascular mortality among patients on dialysis.

## Introduction

Chronic kidney disease (CKD) is a global public health concern^1^. End-stage renal disease (ESRD) is a chronic and progressive decline in kidney function. A substantial number of CKD patients progress to ESRD and impose an enormous health and economic burden^2^. More than two million people suffer from ESRD worldwide^3^. Renal replacement therapy with maintenance hemodialysis or chronic peritoneal dialysis is increasingly used in the care of patients with ESRD^4^. Given that patients with ESRD undergoing dialysis have a substantial risk of mortality^5,6^, the risk factors for mortality in this population should be identified.

Alkaline phosphatase (ALP) is a hydrolase enzyme that catalyzes phosphate from nucleotides and proteins^7^. ALP usually originates from the liver or bone and concentrates in the bone, liver, placenta, and kidney. Several^8–18^ but not all^19,20^ epidemiologic studies reported that elevated serum levels of ALP are associated with increased all-cause mortality among patients on hemodialysis and peritoneal dialysis. Nonetheless, for cardiovascular mortality, studies^10,14,15,20^ have yielded contradicting results. Meanwhile, the risk estimates of the association vary widely.

Previous meta-analyses did not assess the effect of serum ALP levels on subsequent mortality risk among patients on dialysis. Given the varied and conflicting findings in the published studies, we conducted this meta-analysis to investigate whether baseline serum levels of ALP are an independent predictor of cardiovascular or all-cause mortality in patients with ESRD on hemodialysis or peritoneal dialysis.

## Results

### Search results and study characteristics

**Figure 1:**
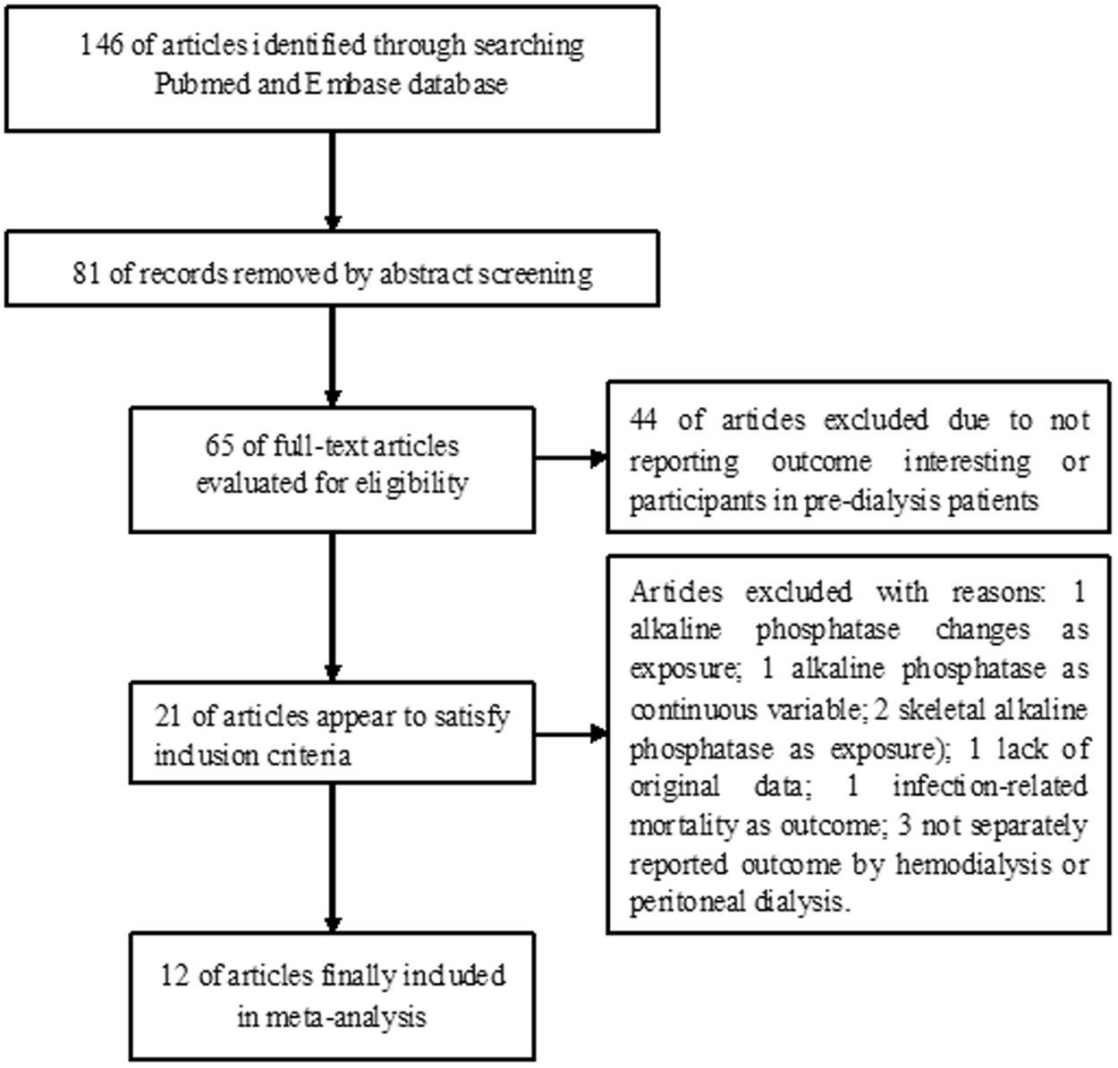
Flowchart of the study selection process.

A flowchart of the study selection process is presented in Fig. 1. In brief, 146 articles were retrieved in the initial literature searches after removing duplicates. Subsequently, 134 articles were excluded after applying our predefined inclusion criteria. Thus, 12 studies^8–17,20,21^ with 393,200 patients on dialysis were finally included in the meta-analysis. Table 1 presents a summary of the general characteristics of the included studies. These included studies were mainly conducted in the United States^8–10,12,13^, mainland China^14^, Japan^11,15^, South Africa^17^, and Taiwan^16,20,21^. One study^10^ was a retrospective analysis of a randomized controlled trial, and others were retrospective cohort designs. Eight studies^8–11,15–17,20^ enrolled patients on hemodialysis, three studies^13,14,21^ enrolled patients on peritoneal dialysis, and one study^12^ comprised both patients on hemodialysis and peritoneal dialysis. Individual study sample sizes varied from 90 to 185,277, and the follow-up duration ranged from 1.0 year to 7.0 years. The mean reported age of patients was between 47.5 and 66 years. Six studies^8,9,12,15,16,20^ with 7 to 8 Newcastle–Ottawa Scale (NOS) stars were grouped as good quality, and the others^10,11,13,14,17,21^ achieved 5–6 stars.

**Table 1:** Summary of clinical studies included in the meta-analysis.

Study/year	Region	Design	Type of patients	Sample size (%men)	Age/range Mean (SD)	Comparison	Events number/ OR or HR (95% CI)	Follow-up (year)	Adjustment for Covariates	Total NOS
Regidor *et al*. 2008^8^	USA	Retrospective study	HD	73,960 (53.6)	61.1 ± 15.6	Higher vs. lower ≥120 U/L vs. <120 U/L	Total death: 251 1.25(1.21–1.29)	3	Age, gender, race, ethnicity, DM, smoking, dialysis vintage, primary insurance, marital status, Kt/V, dialysis catheter, residual renal function during the entry, AST, ALT, and PTH.	7
Abramowitz *et al*. 2010^9^	USA	Retrospective cohort study	HD	10,743 (36)	51.4 ± 15.8	Quartile 4 vs. 1; ≥104 U/L vs. ≤66U/L	Total death: (949) 1.65 (1.36–2.01)	6.8	Age, gender, race/ethnicity, DM, hypertension, CVD; insurance; hospitalization within 28 days after index date; eGFR, corrected calcium, serum albumin, hemoglobin, TC, bicarbonate, AST, and bilirubin	8
Beddhu *et al*. 2010^10^	USA	Retrospective analysis of RCT	HD	1,827 (56)	58 ± 14	Higher vs. lower ≥97 IU/L vs. <97 IU/L	Total death: 871 1.20 (1.01–1.43) CV death: 408 0.94 (0.73–1.22)	6.6	Age, gender, race, Kt/V and flux interventions, clinical center, dialysis years, type of vascular access, comorbidity, hematocrit, albumin, AST, ALT, serum calcium, phosphorus and PTH	6
Yamashita *et al*. 2011^11^	Japan	Retrospective cohort study	HD	195 (61)	62.1 ± 12.3	Higher vs. lower ≥236 IU/L vs. <236IU/L	Total death: 68 2.49 (1.34 –4.58)	5	Age, gender, dialysis months, CAD, cerebrovascular disease, PVD, DM, BMI, Hb, serum albumin, AST, calcium, phosphorus and PTH levels.	5
Rhee *et al*. 2013^12^	USA	Retrospective study	PD and HD	108,567 (54)	59 ± 17	Highest vs. reference lower ≥120 U/L vs. 70 to <90 U/L	Total death: 5605 1.91(1.68–2.16); PD 1.62 (1.51–1.74); HD	2.7	Age, sex, race/ethnicity, DM, CHF, AHD, PVD, CVD, tobacco, dialysis vintage, insurance, marital status, BMI, ferritin, WBC, albumin, total iron binding capacity, bicarbonate, creatinine, lymphocyte, nPCR, calcium, phosphorus, Hb, and erythropoiesis stimulating agent.	7
Fein *et al*. 2013^13^	USA	Retrospective study	PD	90 (49)	52 ± 16	Higher vs. lower ≥120 U/L vs. <120 U/L	Total death: NR 6.00 (1.19–30.3)	2.61	Age, race, sex, DM, hypertension, dialysis vintage at enrollment, albumin, corrected calcium, PTH, creatinine, BUN, Hb, iron, AST, and WBC	5
Liu *et al*. 2014^14^	China	Retrospective cohort study	PD	1,021 (59.1)	47.5 ± 15.5	Quartile 4 vs. 1; ≥82 U/L vs. ≤52 U/L	Total death: 203 2.05 (1.24–3.41) CV death: 109 2.40 (1.20–4.78)	2.58	Age, sex, 24-h urine output, BP, comorbidity score, hemoglobin, albumin, ALT, AST, phosphate binders use, physiologic calcium peritoneal dialysate use, corrected calcium, phosphorus, and iPTH.	6
Maruyama *et al*. 2014^15^	Japan	Retrospective cohort study	HD	185,277 (61.9)	66 ± 12	Quartile 4 vs. 1; ≥309 U/L vs. ≤183 U/L	Total death: 14,230 1.46 (1.33–1.60) CV death: 6,396 1.25 (1.10–1.42)	1	Age, sex, dialysis duration, BMI, underlying disease, comorbid disease, medication, albumin; BUN, creatinine, CRP, Hb, corrected calcium, phosphorus, magnesium, and iPTH.	7
Chang *et al*. 2014^16^	Taiwan	Retrospective study	HD	9,514 (46)	61.7 ± 13.4	Quintile 5 vs. quintile 1; > 150 U/L vs. <60 U/L	Total death: 3,507 1.58 (1.41–1.76)	3.2	Age, sex, DM, dialysis vintage, hematocrit, phosphorus, calcium, iPTH, albumin, creatinine, BUN, nPCR, dialysis dose, ALT, glucose, UA, TC, TG, and ferritin	8
Zhu *et al*. 2016^20^	Taiwan	Retrospective study	HD	1,126 (46.4)	60.0 ± 12.3	Quartile 4 vs. 1; ≥104 U/L vs. ≤66 U/L	Total death: 229 1.00 (0.65–1.54) CV death: 45 0.71 (0.25 –2.08)	5	Age, sex, dialysis vintage, etiology of renal failure, use of EPIAO, vitamin D, antihypertensive drug, or iron, parathyroidectomy, WBC, albumin, Hb, TC, TG, blood glucose, AST, ALT, bilirubin, corrected calcium, UA, ferritin, iPTH, Kt/V, and cardiac-thoracic ratio.	7
Waziri *et al*. 2017^17^	South Africa	Retrospective study	HD	213 (64)	54.5 ± 15.6	Higher vs. lower ≥112 U/L vs. <112U/L	Total death: 55 2.50 (1.24–5.01)	7	Age, phosphate, calcium, PTH, DM, SBP, 25(OH)D, AST, albumin, and serum calcium.	6
Liu *et al*. 2017^21^	Taiwan	Retrospective study	PD	667 (42.9)	52.2 ± 13.9	Quartile 4 vs. 1; ≥119 U/L vs. ≤62 U/L	Total death: 65 1.88 (0.89–3.98) CV death: 8 2.23 (0.18–27.8)	2.72	Age, sex, 24-hour urinary volume, Hb, albumin, AST and ALT, Ca, phosphate and iPTH.	6

Abbreviations: HD, hemodialysis; PD, peritoneal dialysis; NR, not reported; OR, odds ratio; HR, hazard ratio; CI, confidence interval; RCT, randomized controlled trial; DM, diabetes mellitus; BMI, body mass index; TC, total cholesterol; TG, triglyceride; SBP, systolic blood pressure; AST, aspartate aminotransferase; ALT, alanine aminotransferase; ALP, alkaline phosphatase; CRP, C-reactive protein; nPCR, normalized protein catabolic rate; WBC, white blood cell; CVD, cardiovascular disease; CHF, congestive heart failure; PVD, peripheral vascular disease; AHD, arteriosclerotic heart disease; Hb, hemoglobin; BUN, blood urea nitrogen; iPTH, intact parathyroid hormone; UA, uric acid; eGFR, estimated glomerular filtration rate; NOS, Newcastle-Ottawa Scale.

### Association of serum ALP and all-cause mortality

**Figure 2:**
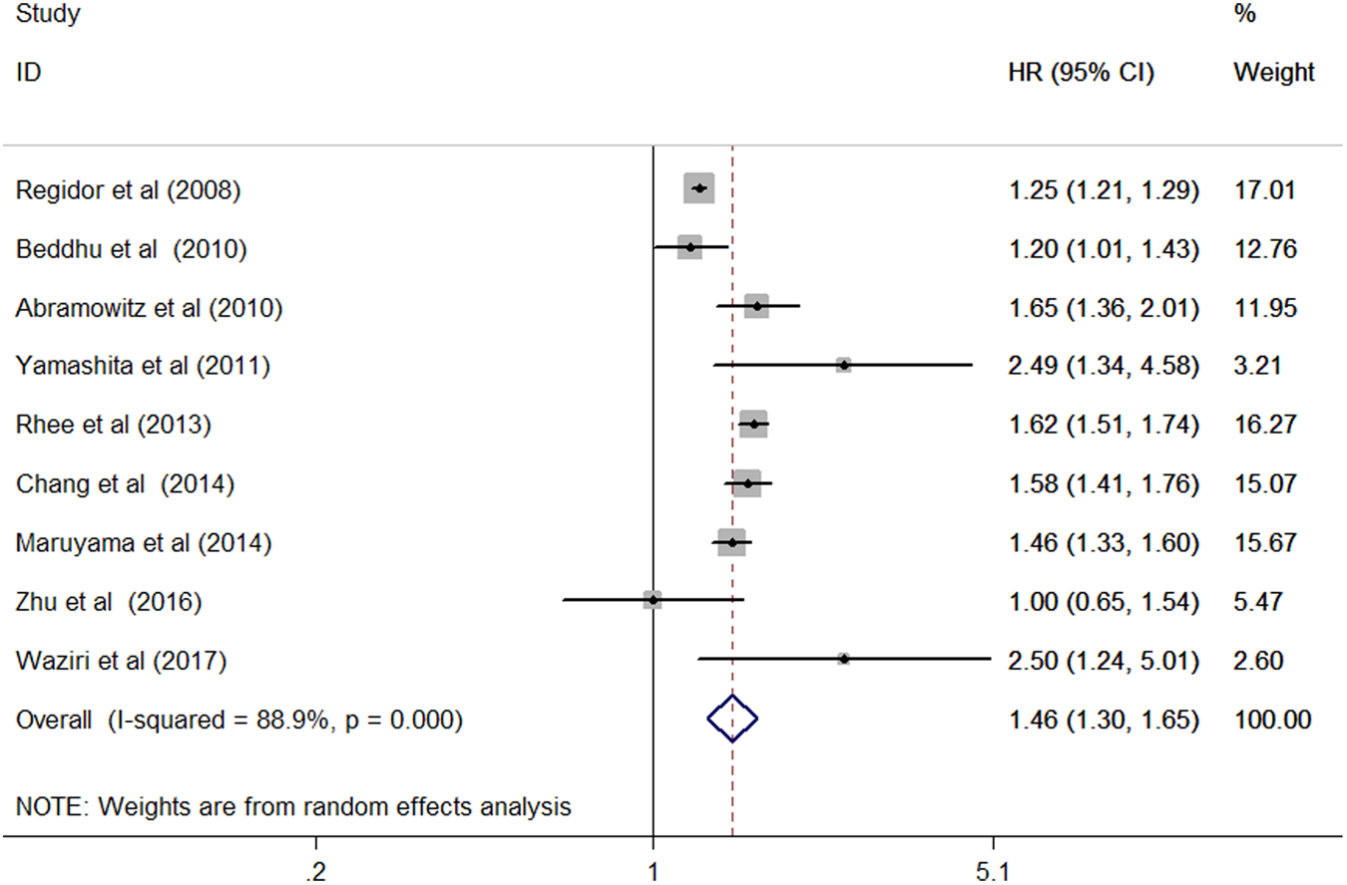
Forest plots showing HR and 95% CI of all-cause mortality among hemodialysis patients comparing the highest to the reference lower serum alkaline phosphatase.

**Figure 3:**
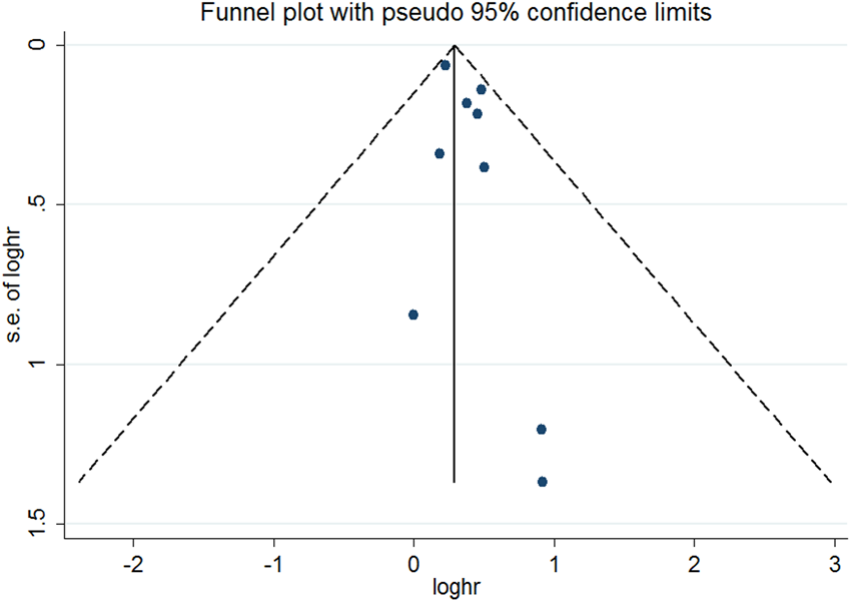
Funnel plot of serum alkaline phosphatase and all-cause mortality risk among hemodialysis patients.

Nine included studies^8–12,15–17,20^ investigated the association of serum ALP with all-cause mortality among patients on hemodialysis. As shown in Fig. 2, elevated serum ALP levels were associated with increased all-cause mortality (HR 1.46; 95% CI 1.30–1.65) in the random effect model compared with the reference low serum ALP. Substantial heterogeneity (*I* ^2^ = 88.9%; *p* < 0.001) was observed among the included studies. Evidence of publication bias was not found as determined by the Begg’s test (*p* = 0.754), Egger’s test (*p* = 0.147), and funnel plot (Fig. 3). Stratified analyses indicated that associations were consistently observed between elevated serum ALP levels and all-cause mortality risk in each predefined subgroup (Table 2).

**Table 2:** Subgroup analyses of serum alkaline phosphatase with all-cause mortality among hemodialysis patients.

Subgroup	No. of studies	Pooled HR	95%CI	Heterogeneity between studies
**Sample sizes**				
≥2,000	5	1.49	1.30–1.72	p < 0.001; I^2^ = 93.5%;
<2,000	4	1.69	1.12–2.45	p = 0.020; I^2^ = 69.4%
**Mean age**				
≥60 years	6	1.46	1.26–1.69	p < 0.001; I^2^ = 92.1%;
<60 years	3	1.53	1.11–2.09	p = 0.015; I^2^ = 76.1%
**Follow-up duration**				
≥3 years	7	1.43	1.23–1.67	p < 0.001; I^2^ = 81.4%
<3 years	2	1.54	1.40–1.71	p = 0.080; I^2^ = 67.3%
**Region**				
No-Asian	5	1.45	1.22–1.73	p < 0.001; I^2^ = 92.4%
Asian	4	1.50	1.29–1.73	p = 0.066; I^2^ = 58.2%
**Alkaline phosphatase levels**				
Cutoff analysis	4	1.37	1.12–1.67	p = 0.032; I^2^ = 66.0%
Quartile/tertile analysis	5	1.55	1.43–1.67	p = 0.111; I^2^ = 46.8%
**Overall NOS**				
≥7	6	1.45	1.27–1.66	p < 0.001; I^2^ = 92.1%
<7	3	1.82	1.02–3.26	p = 0.015; I^2^ = 76.4%
**Adjustment of liver function**				
Yes	7	1.43	1.23–1.67	p < 0.001; I^2^ = 81.4%
No	2	1.54	1.40–1.71	p = 0.080; I^2^ = 67.3%

HR, hazard ratio; CI, confidence interval; NOS, Newcastle-Ottawa Scale. ^#^Liver function markers includes aspartate aminotransferase and alanine aminotransferase.

**Figure 4:**
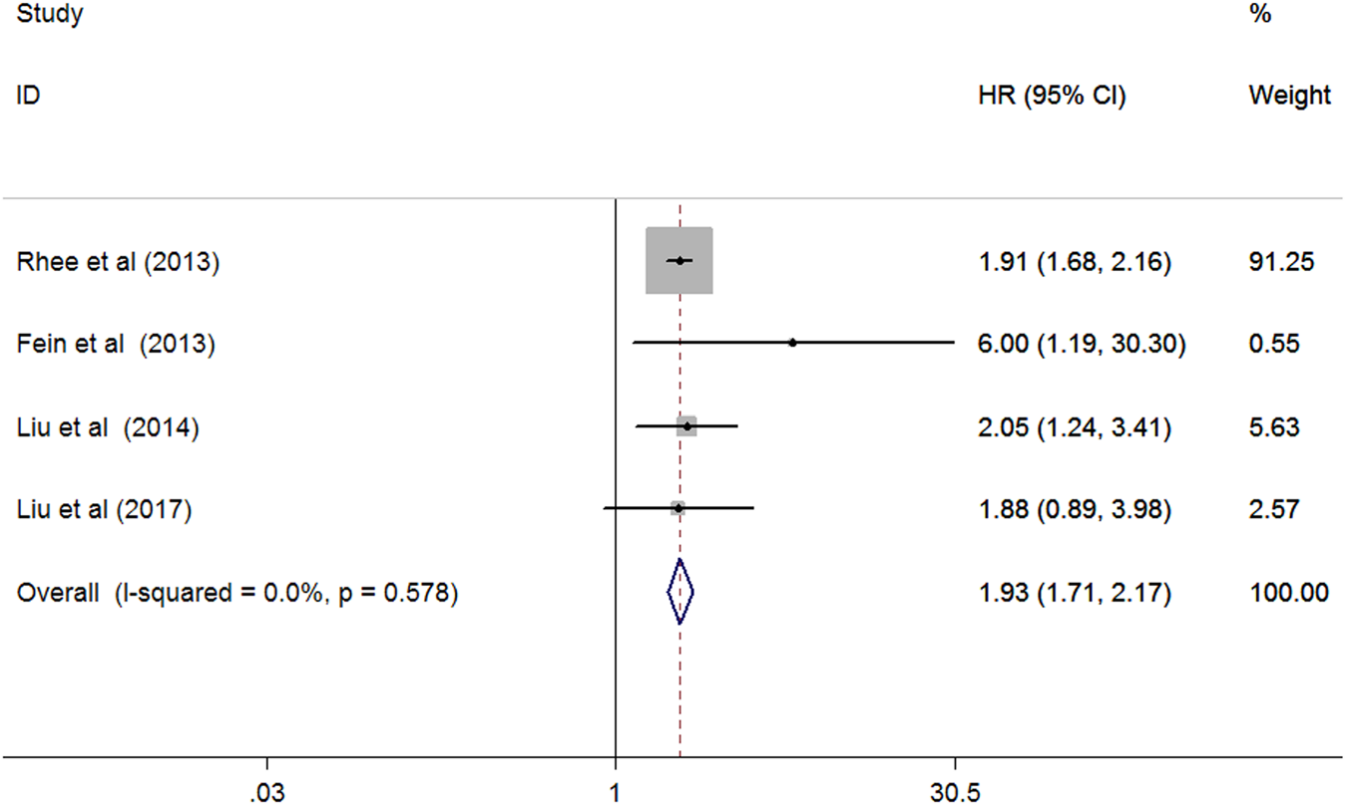
Forest plots showing HR and 95% CI of all-cause mortality among peritoneal dialysis patients comparing the highest to the reference lower serum alkaline phosphatase.

Four studies^12–14,21^ investigated the association of serum ALP with all-cause mortality among patients on peritoneal dialysis. As shown in Fig. 4, the pooled HR for all-cause mortality was 1.93 (95% CI 1.71–2.17) when the highest was compared with the reference low serum ALP levels in a fixed-effect model, and no heterogeneity was found across studies (*I* ^2^ = 0.0%; *p* = 0.578).

### Association of serum ALP and cardiovascular mortality

**Figure 5:**
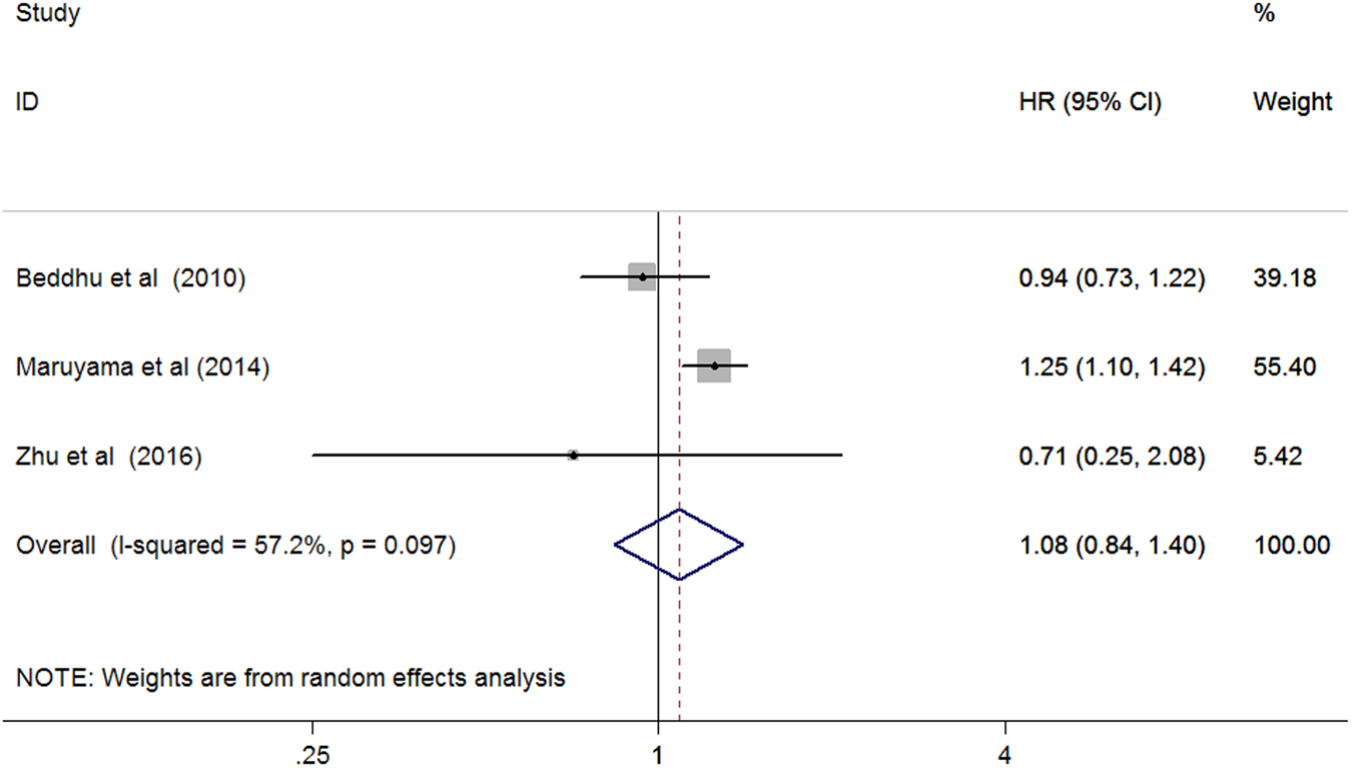
Forest plots showing HR and 95% CI of cardiovascular mortality among hemodialysis patients comparing the highest to the reference lower serum alkaline phosphatase.

Three studies^10,15,20^ assessed the association of serum ALP with cardiovascular mortality among patients on hemodialysis. As shown in Fig. 5, elevated serum ALP levels were not associated with increased cardiovascular mortality (HR 1.08; 95% CI 0.84–1.40) in a random effect model compared with the reference low serum ALP with substantial heterogeneity across studies (*I* ^2^ = 52.7%; *p* = 0.097). Sensitivity analyses by removal of any study at a time did not change the direction of the pooled effect size (data not shown).

**Figure 6:**
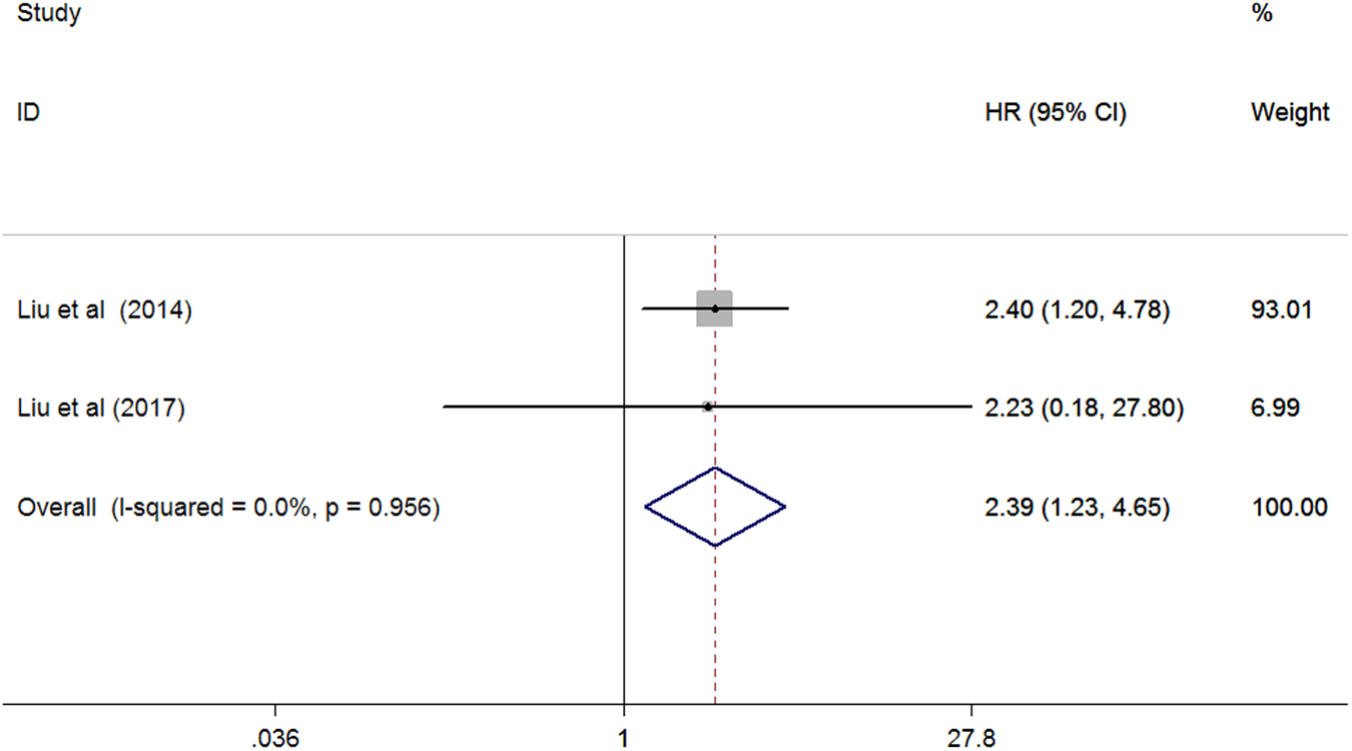
Forest plots showing HR and 95% CI of cardiovascular mortality among peritoneal dialysis patients comparing the highest to the reference lower serum alkaline phosphatase.

Two studies^14,21^ reported cardiovascular mortality as an outcome among patients on peritoneal dialysis. As shown in Fig. 6, elevated serum ALP levels significantly increased cardiovascular mortality (HR 2.39; 95% CI 1.23–4.65) in a fixed-effect model compared with the reference low serum ALP, and substantial heterogeneity was found across studies (*I* ^2^ = 0%; *p* = 0.956).

## Discussion

This study is the first meta-analysis to evaluate the association between serum ALP and risk of cardiovascular and all-cause mortality in patients on dialysis. The main finding of the current meta-analysis showed that elevated serum ALP levels were associated with an increased all-cause mortality risk in patients on dialysis even after adjustment of liver enzymes and bone metabolism parameters. In addition, elevated serum ALP levels appeared to significantly increase cardiovascular mortality among patients on peritoneal dialysis. However, no clear effect was indicated on cardiovascular mortality risk prediction among patients on hemodialysis.

Circulating ALP levels often increase in ESRD. In this study, the observed all-cause mortality risk was more pronounced among patients on peritoneal dialysis than among patients on hemodialysis. Patients on hemodialysis with the highest serum ALP levels significantly increased 46% risk of all-cause mortality. Alternatively, patients on peritoneal dialysis exhibiting the highest serum ALP levels were associated with 93% risk of all-cause mortality. In addition, the association was more pronounced among studies with a short follow up than studies with a long follow-up duration. One-year mortality was 19.8% among 385,074 patients on hemodialysis^22^. The presence of bone and liver diseases may affect the association of serum ALP with mortality risk. However, the association was still observed in the studies even after adjustment for liver function tests and serum levels of parathyroid hormone, phosphorus, and calcium. Therefore, serum ALP levels should be considered as an independent risk factor for all-cause mortality.

Cardiovascular disease is the main cause of death in patients receiving dialysis^23,24^. However, the association between serum ALP levels and cardiovascular mortality risk in patients on hemodialysis is unclear. Our meta-analysis indicated that elevated serum ALP levels appeared to significantly increase cardiovascular mortality among patients on peritoneal dialysis but not in patients with hemodialysis. When serum ALP was used as a time-varying exposure variable, high (≥97 IU/l) versus low ALP (<97 IU/l) was associated with a 34% higher risk of cardiovascular mortality^10^. This finding suggested that the effect of ALP, which leads to increasing cardiovascular death, was time-dependent.

Serum ALP is primarily used as an indicator for hepatic and bone disease. Apart from liver and bone diseases, serum ALP levels are elevated in various cancers, chlorpropamide therapy, hormonal contraception, pregnancy, and hyperthyroidism^25^. Our findings were in line with evidence from a previous meta-analysis^26^; elevated serum levels of ALP indicated a high all-cause mortality in people with normal or preserved renal function. Moreover, elevated serum bone-specific ALP was also associated with mortality risk in patients on hemodialysis^19,27^.

The exact mechanisms for the association of alkaline phosphatase with mortality risk remain unclear. A possible explanation for the observed association is that ALP is a marker of high-turnover bone disease^28^. ALP can promote vascular calcification by hydrolyzing pyrophosphate in the arterial wall^29–31^. In addition, inflammation may be another potential mechanism for the association between high serum ALP levels and increased mortality^32^.

Several potential limitations should be mentioned in this meta-analysis. First, substantial heterogeneity was observed among studies involving patients on hemodialysis. However, substantial heterogeneity did not obviously disappear in the subgroup analysis. The observed heterogeneity may be correlated with patient characteristics and dialysis regimen. Second, serum ALP levels were determined at a single time, and misclassification in ALP categories was not excluded. Third, a “U”-shaped correlation between all-cause mortality and serum levels of ALP was reported in patients on hemodialysis. Low ALP was associated with a high risk of all-cause mortality^12^. Thus, selecting the lowest serum ALP as a reference value may have underestimated the actual risk estimate. Finally, all the included studies were retrospective analyses of an existing database, and more prospective cohort studies are needed to confirm this association.

Elevated serum ALP was an independent risk factor for all-cause mortality among patients on hemodialysis or peritoneal dialysis. Our findings revealed that patients on dialysis with elevated serum ALP were candidates at high risk of all-cause mortality, and low ALP levels may reduce all-cause mortality rates in the dialysis population. However, a U-shaped association of serum ALP with mortality risk in patients on dialysis needs to further investigated. Moreover, future well-designed prospective studies are necessary to investigate the association between elevated serum ALP and cardiovascular mortality among patients on dialysis.

## Methods

### Data sources and search strategy

This meta-analysis was performed and reported following the standard criteria of the Meta-analysis Of Observational Studies in Epidemiology statement^33^. A comprehensive literature search was conducted using the PubMed and Embase databases from inception to March 2017. Key words used for the search were (alkaline phosphatase) AND (end-stage renal disease OR renal replacement therapy OR hemodialysis OR peritoneal dialysis) AND (death OR mortality) AND (follow-up OR longitudinal). Language restrictions were not applied in the electronic literature searches. To identify additional eligible studies, we manually reviewed the reference lists of relevant articles.

### Study selection

Studies were included according to the following inclusion criteria: (1) prospective or retrospective cohort studies; (2) inclusion of patients with ESRD undergoing hemodialysis or peritoneal dialysis; (3) baseline serum ALP levels as exposure; and (4) provided multiple adjusted odds ratio (OR) or hazard ratio (HR) and 95% confidence interval (CI) of cardiovascular or all-cause mortality comparing the highest with the reference lower serum ALP levels. Exclusion criteria were (1) pre-dialysis CKD patients; (2) skeletal ALP as exposure; (3) time-varying serum ALP as exposure; and (4) risk estimates were not reported separately for patients on hemodialysis or peritoneal dialysis.

### Data collection and quality assessment

The following items were extracted from the included articles by two independent authors: first author’s surname, publication year, origin of study, study design, sample size, type of dialysis, mean age of patients, male gender proportion, cutoff value of ALP comparison, number of death events, multivariate adjusted risk estimates for all-cause or cardiovascular mortality, follow-up period, and adjustment variables. To assess the quality of the included studies, Newcastle-Ottawa Scale (NOS) for cohort studies^34^ was used to evaluate the methodological quality. The following three aspects were assessed: selection of study participants, comparability of groups, and ascertainment of outcomes. Using this scale, the maximum score was 9 stars. Studies were graded as good quality if they achieved a score of ≥7 stars. Disagreements in data collection and quality assessment were resolved through consensus.

### Data synthesis and analysis

All the meta-analyses were performed using STATA software (version 12.0). The pooled multivariable-adjusted HR and 95% CI of cardiovascular or all-cause mortality was computed for the highest versus the reference low category of serum ALP levels. Statistical heterogeneity across studies was assessed using the Cochrane *Q* test and *I* ^2^ statistic. The significance of the statistical heterogeneity was set at the *I* ^2^ statistic ≥50% and/or Cochrane *Q* test *p* < 0.10. We selected a random effect model for pooling risk estimates if significant statistical heterogeneity was present; otherwise, a fixed-effect model was utilized. Subgroup analyses were planned for patient types (hemodialysis versus peritoneal dialysis), region (Asia versus no-Asia), sample size (≥2000 versus <2000), mean age (≥60 versus <60), comparison of ALP levels (single cutoff versus ≥3 category analysis), follow-up duration (≥3 years versus <3 years), and NOS stars (≥7 versus <7). Publication bias was assessed using the Begg’s test, Egger’s test, and a funnel plot.
